# Author Correction: Strategies to package recombinant Adeno-Associated Virus expressing the N-terminal gasdermin domain for tumor treatment

**DOI:** 10.1038/s41467-022-29454-7

**Published:** 2022-05-19

**Authors:** Yuan Lu, Wenbo He, Xin Huang, Yu He, Xiaojuan Gou, Xiaoke Liu, Zhe Hu, Weize Xu, Khaista Rahman, Shan Li, Sheng Hu, Jie Luo, Gang Cao

**Affiliations:** 1 State Key Laboratory of Agricultural Microbiology, Hubei Hongshan Laboratory, Huazhong Agricultural University, 430070 Wuhan, China; 2 College of Veterinary Medicine, Huazhong Agricultural University, 430070 Wuhan, China; 3 College of Biomedicine and Health, Huazhong Agricultural University, 430070 Wuhan, China; 4 Department of Medical Oncology, Hubei Cancer Hospital, Huazhong Agricultural University, Wuhan, China; 5 Taihe Hospital, Hubei University of Medicine, Shiyan, 442000 Hubei China

**Keywords:** Cancer immunotherapy, Cancer immunotherapy, Cell death

Correction to: *Nature Communications* 10.1038/s41467-021-27407-0, published online 09 December 2021.

This Article contained an error in the Introduction and Discussion sections. The original version of the Article omitted a reference to a study that used a strategy to drive the expression of GSDM^NT^ in rAAV by a Schwann-cell-specific promoter. The reference has now been included (new reference 18) and the Introduction and Discussion sections have been revised accordingly.

In the Introduction, the reference to this study has been added and the content of the second paragraph of the Introduction following the sentence “Moreover, rAAV has strong penetrability to solid tumors and has already been applied in tumor therapies^15,16^.” has been revised accordingly. In particular, the original sentence “However, GSDM^NT^ is highly toxic to the cells due to its strong pore-forming activity, thus it is extremely difficult to biologically produce GSDM^NT^ or package it on a large scale^17^.” now reads “However, GSDM^NT^ is highly toxic to the cells^17^; its intracellular expression can lead to pyroptosis during AAV packaging and thus poses a challenge to delivery GSDM^NT^ by AAV.” The following new sentence has been added that reads “While it is a good strategy to drive the expression of GSDM^NT^ in rAAV by cell-specific promoter, as it has been successfully demonstrated with a Schwann-cell-specific promoter for Schwannoma cancer therapy^18^, some cell-specific promoters cannot completely avoid the leakage expression of GSDM^NT^ during AAV packaging process, which may affect the titer of the virus.”

In the first paragraph of the Discussion, following the sentence that reads "However, owing to the extremely high cytotoxicity of GSDM^NT^ during packaging, the conventional rAAV packaging approaches are impossible to achieve rAAV-GSDM^NT^ with enough titer for clinical use.”, a new sentence has been added: “A Schwann-cell-specific promoter has been employed to control the expression of GSDMD^NT^ during AAV packaging, which might be specifically used for schwannomas treatment^18^. However, different cell-specific promoters have different levels of leakage expression, which may affect the titer of the virus and increase the cost for the clinical gene therapy.”

Also in the first paragraph of the Discussion, following the original sentence “In this study, we developed two strategies and successfully packaged rAAV-GSDM^NT^, which can be potentially applied for antitumor therapy.” the following sentences have been added: “The compatibility between transcription factor and the underlying promoter is pivotal for gene expression. To maximize the divergence between the promoter and the potential transcription factors we employed a mammalian promoter and packaged the virus in insect cells, as the insect cell AAV packaging system is well established for large-scale AAV production by fermentation. After screening for several promoters, our data suggested that sf9 insect cells do not express the transcription factors to drive mCBA promoter activity (a promoter that can initiate gene expression in most mammalian cells). Thus, this strategy can produce high titers of rAAV-GSDM^NT^ in sf9 cell that can be used for different types of tumor therapy.”

New reference:

18. Ahmed, S. G., Abdelanabi, A., Doha, M. & Brenner, G. J. Schwannoma gene therapy by adeno-associated virus delivery of the pore-forming protein Gasdermin-D. Cancer Gene Ther 26, 259–267 10.1038/s41417-018-0077-3 (2019).

The remaining reference numbers have also been updated in light of this addition. These errors have been corrected in the HTML and PDF versions of the Article.

