# Supplementary material for: Strategies to package recombinant Adeno-Associated Virus expressing the N-terminal gasdermin domain for tumor treatment
Source: Nat Commun. 2021 Dec 9;12:7155. doi: 10.1038/s41467-021-27407-0 (PMC8660823; doi:10.1038/s41467-021-27407-0)
Supplement: Supplementary file 1 — Supplementary Information [file 41467_2021_27407_MOESM1_ESM.docx]

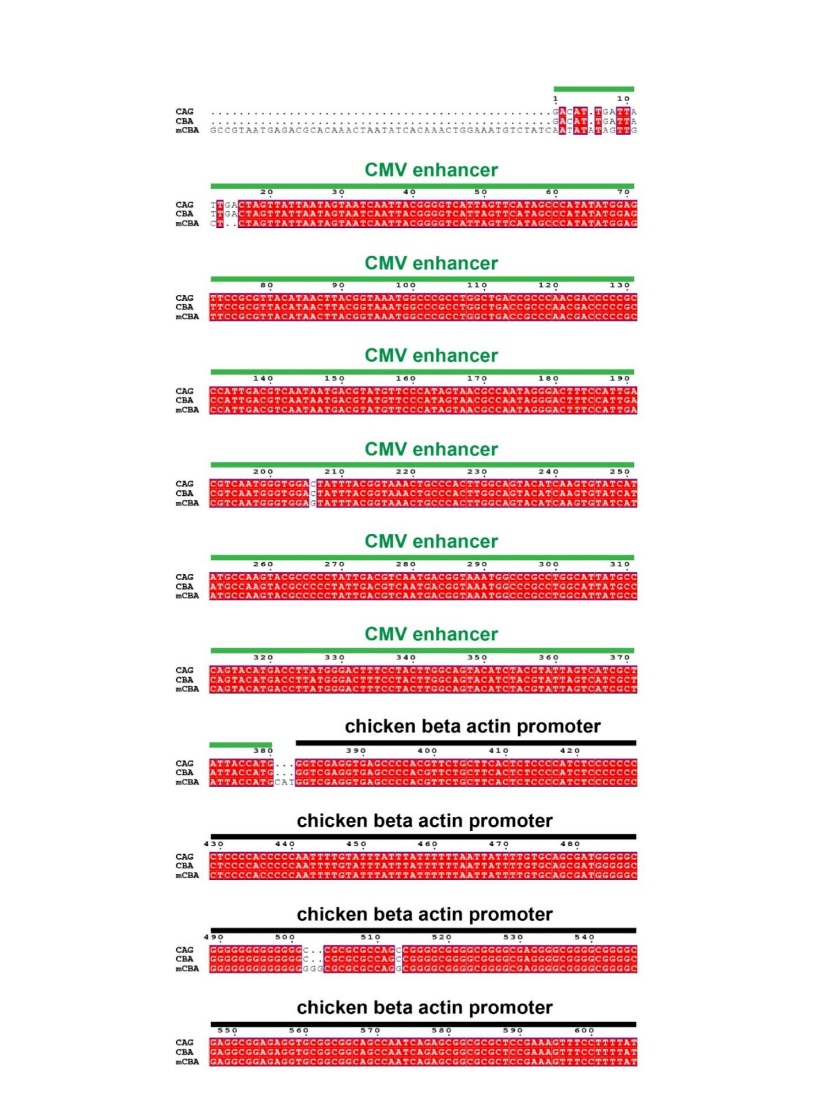

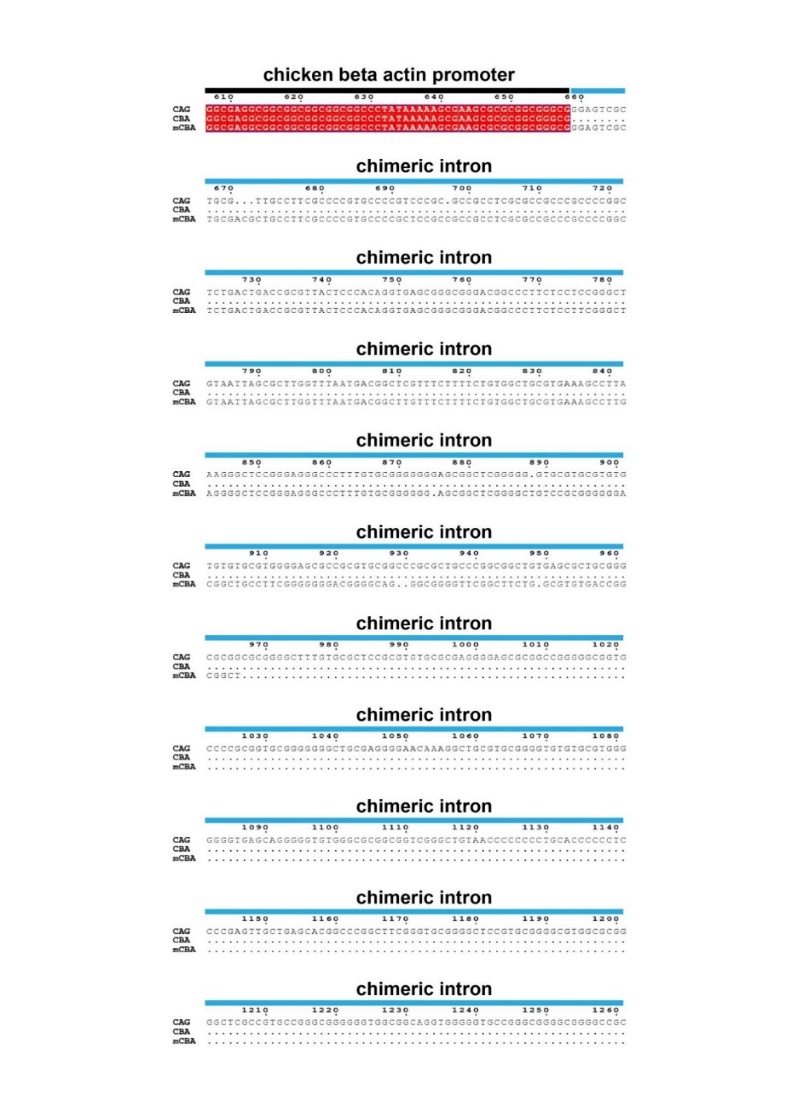

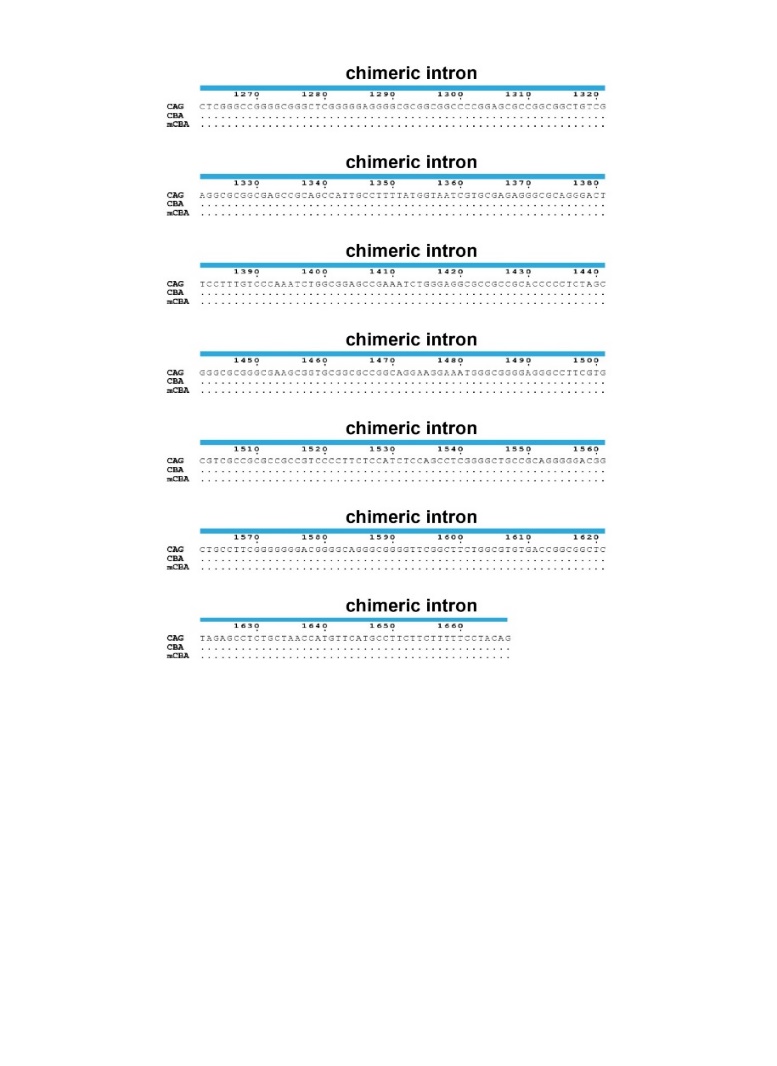


**Supplementary Figure 1. Sequence alignment of CAG, CBA and mCBA promoters.**


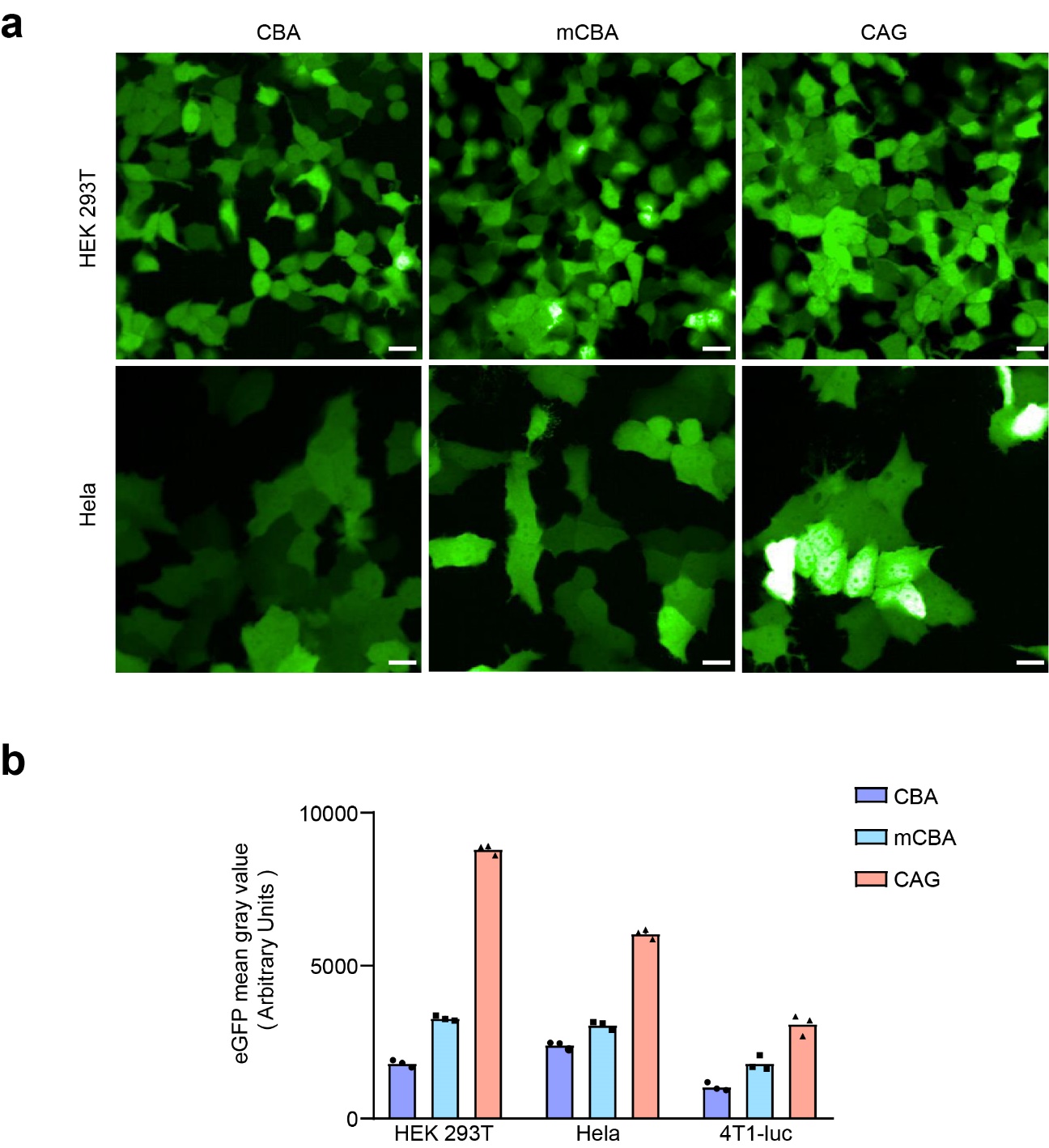


**Supplementary Figure 2.** **Transcription activity assay of CAG, CBA and mCBA promoter activity in HEK 293T, Hela and 4T1-luc cells.** **a,** Representative fluorescence microscopy of HEK 293T and Hela cells showing the expression of eGFP driven by different promoters at 48 h post-transfection. Scale bars, 20 μm. **b,** Quantitative results of the average fluorescence intensity eGFP expression driven by different promoters in HEK 293T, Hela and 4T1-luc cells. All data are representative of three independent experiments. Source data are provided as a Source Data file.


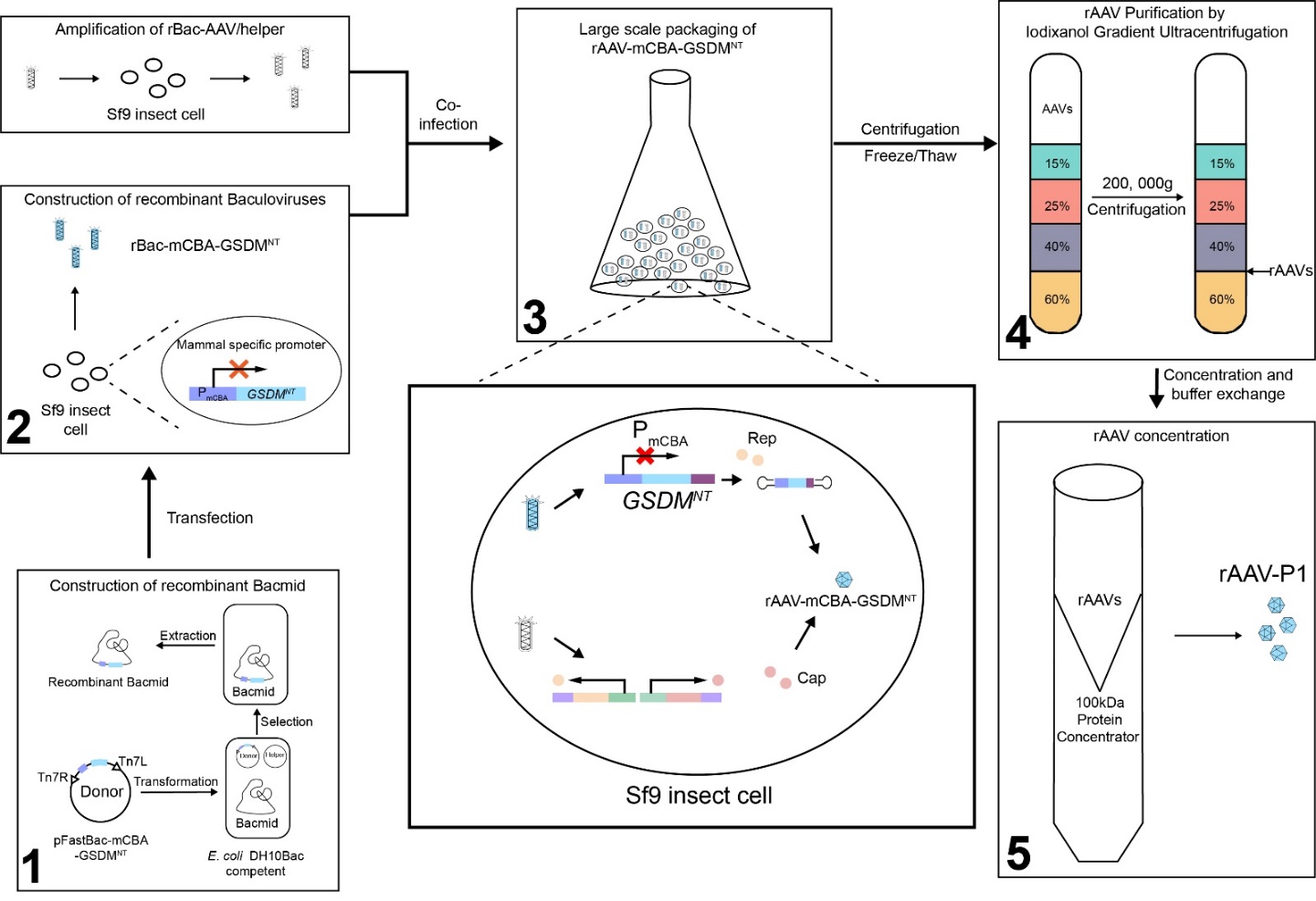


**Supplementary Figure 3. The flow diagram of rAAV-P1 packaging.** 1) Construct Recombinant Donor plasmids carrying ITR-mCBA-GSDM^NT^ and transform it to *E. coli* DH10Bac competent cells. After screening for resistance and blue-white, recombinant Bacmids are obtained. 2) Recombinant Bacmids are transfected into sf9 insect cells to obtain rBac-mCBA-GSDM^NT^ virus. 3) sf9 cells are co-infected with rBac-mCBA-GSDM^NT^ and rBac-AAV/helper to obtain rAAV-mCBA-GSDM^NT^ in large scale. 4) rAAV-mCBA-GSDM^NT^ are centrifuged with iodixanol density gradient. 5) rAAV-P1 are obtained after concentration.


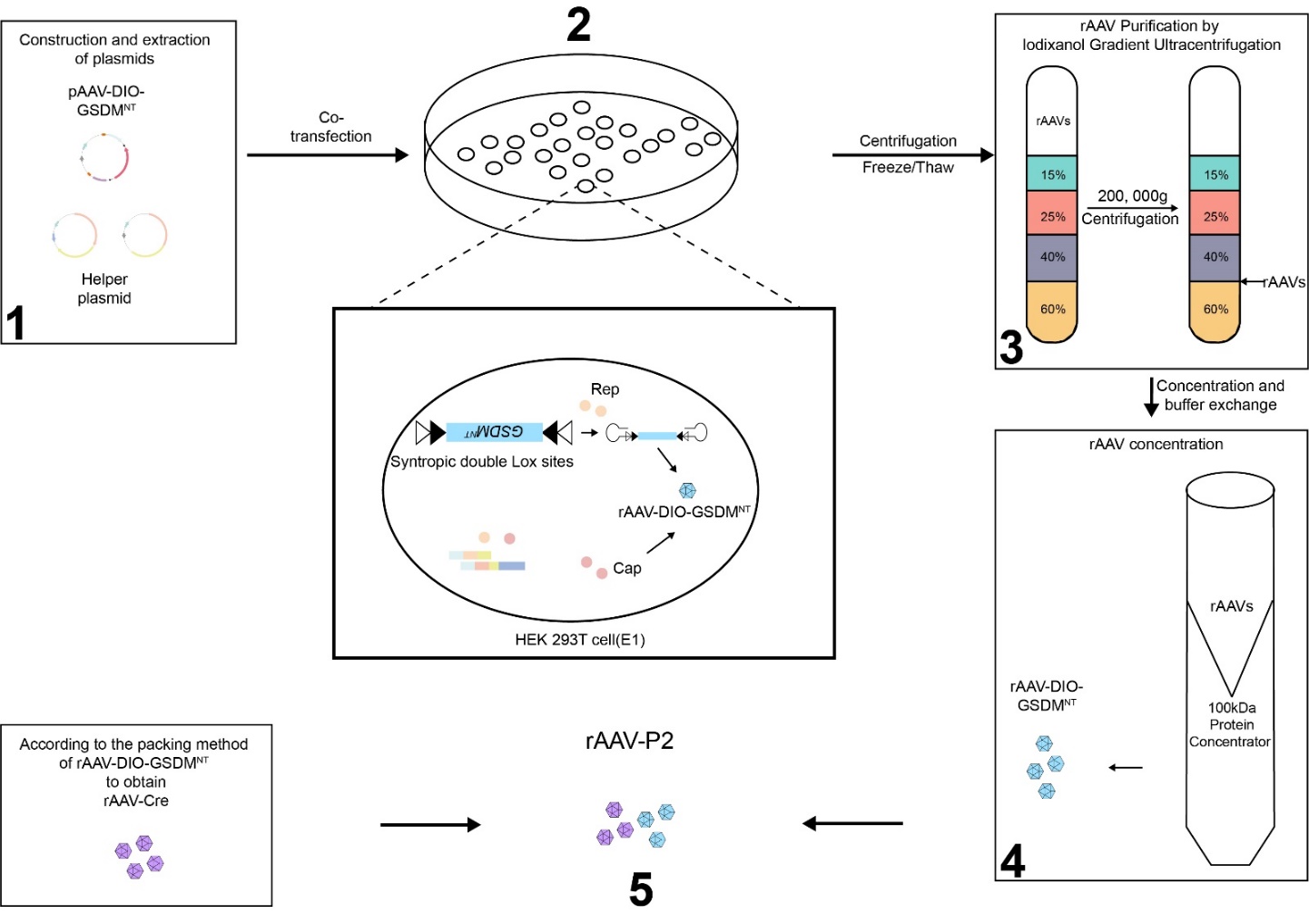


**Supplementary Figure 4. The flow diagram of rAAV-P2 packaging.** 1) Construction of plasmid pAAV-DIO-GSDM^NT^. 2) Co-transfect with pAAV-DJ and pHelper into HEK 293T cells to package rAAV-DIO-GSDM^NT^. GSDM^NT^ is invertedly cloned into rAAV vector flanking with syntropic double Lox sites. 3) After iodixanol density gradient centrifugation and 4) concentration, rAAV-DIO-GSDM^NT^ is obtained. 5) rAAV-Cre is also packaged to revert rAAV-DIO-GSDM^NT^ for GSDM^NT^ expression. In this way, it can totally avoid the expression of GSDM^NT^ during virus packaging.


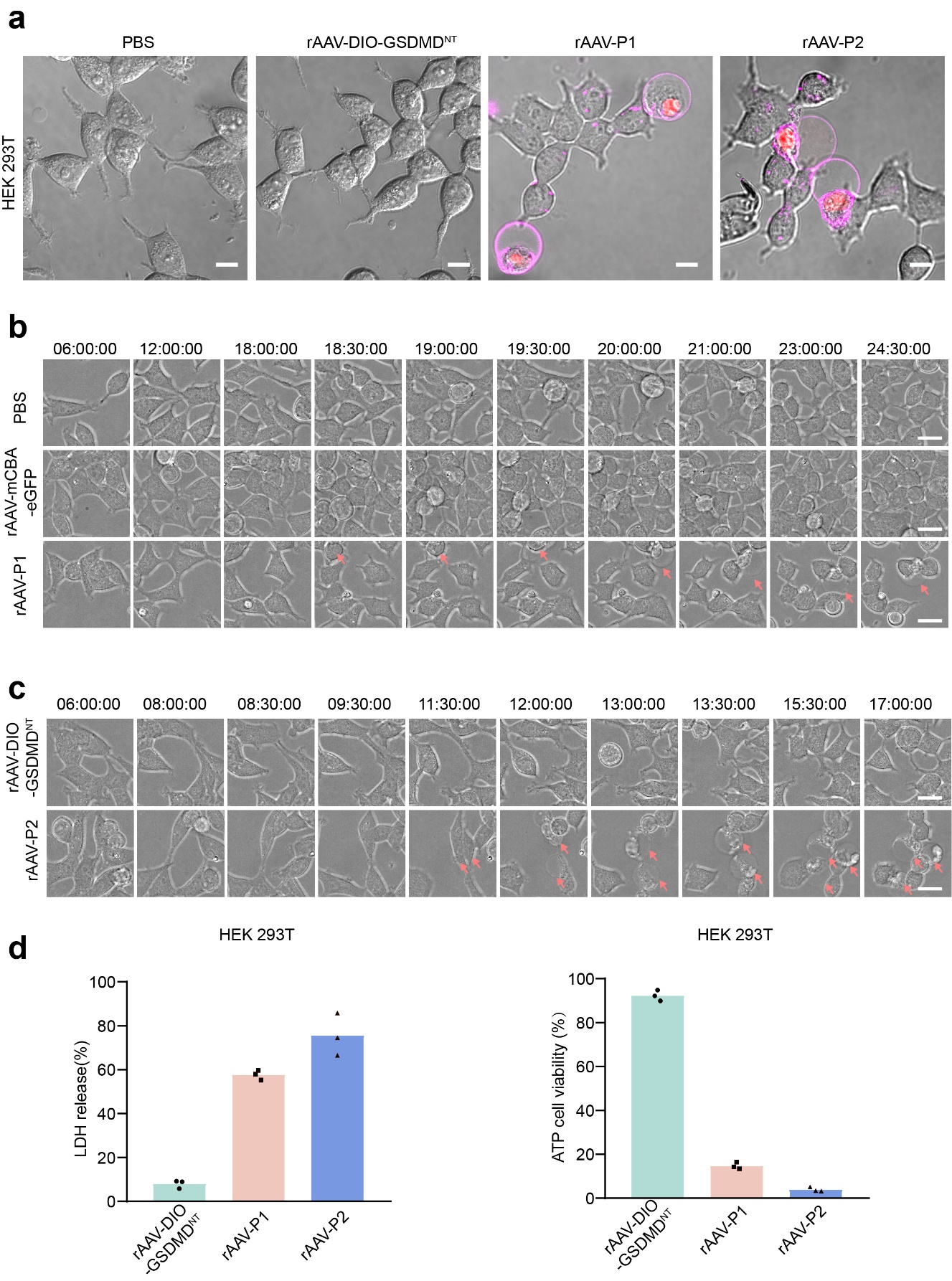


**Supplementary Figure 5. Pyroptosis of HEK 293T cells induced by** **rAAV-P1 and rAAV-P2 treatment.** **a,** Confocal images of the HEK 293T cells treated with rAAVs. Scale bars, 20 μm. The cells were added with Propidium Iodide and Annexin V-APC 15 min before imaging. **b, c,** Confocal high-content cell imaging of HEK 293T cells treated with rAAV-P1 (**b**) and rAAV-P2 (**c**), respectively. Scale bars, 20 μm. **d,** LDH release-based cell death assay and ATP cell viability assay in HEK 293T cells treated with rAAV-DIO-GSDMD^NT^ (rAAV-ef1α-DIO-GSDMD^NT^), rAAV-P1 and rAAV-P2, respectively. Data were expressed as mean ± s.e.m. All date are representative of two independent experiments. Source data are provided as a Source Data file.


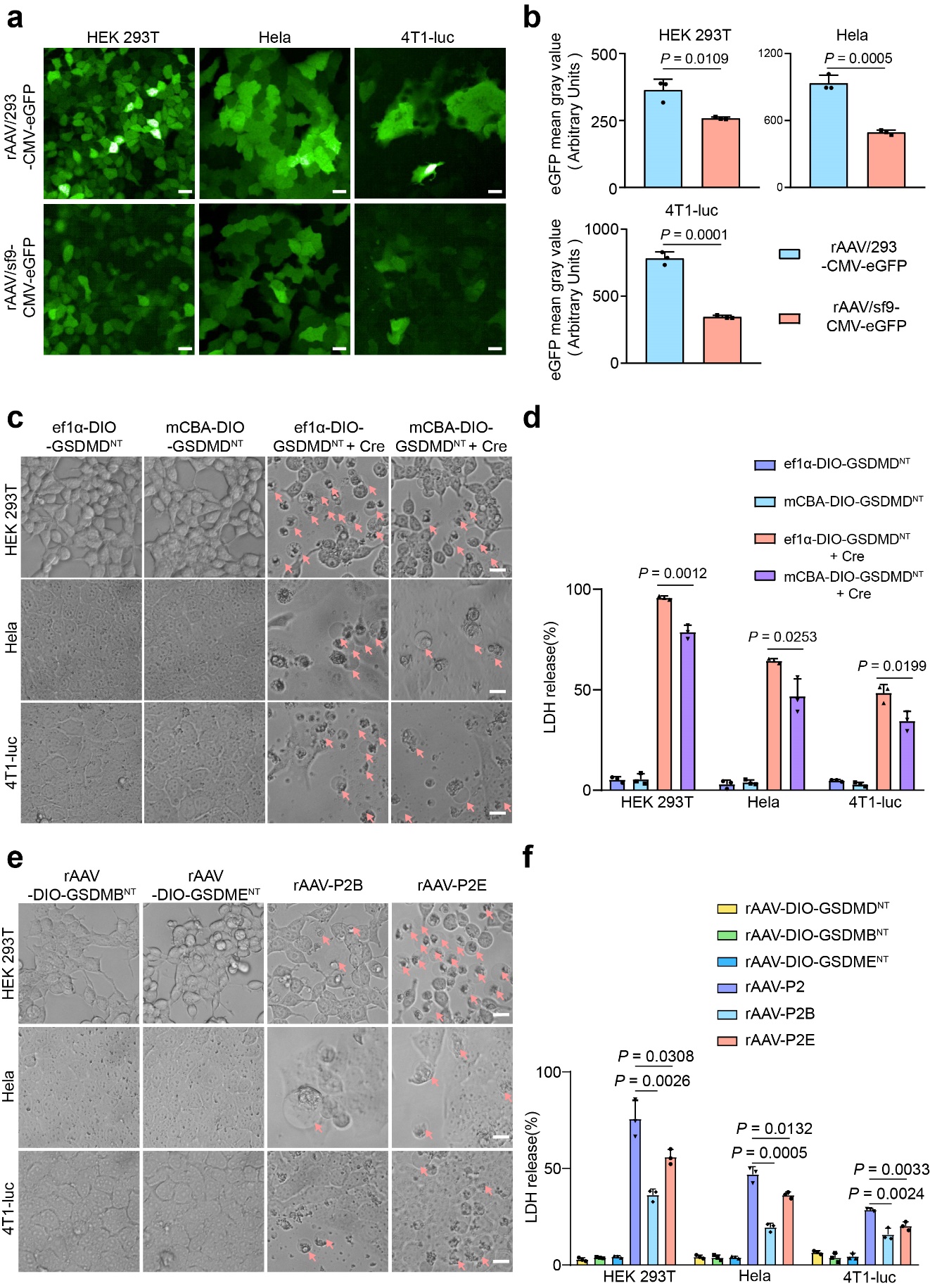


**Supplementary Figure 6. The comparison of induced pyroptosis efficiency between different rAAVs and the versatility of rAAV-P2 packaging strategy. a,** Representative fluorescence microscopy of HEK 293T, Hela, and 4T1-luc cells showing the expression of eGFP transduced by rAAV produced in insect Sf9 cells (rAAV/sf9-CMV-eGFP) and mammalian 293 cells (rAAV/293-CMV-eGFP) 48 hours post infection, respectively. Scale bars, 20 μm. **b,** Quantitative results of the average fluorescence intensity of the eGFP expressed by rAAV/sf9-CMV-eGFP and rAAV/293-CMV-eGFP in HEK 293T, Hela and 4T1-luc cells, respectively. Mean ± s.d., two-tailed unpaired Student’s t-test. **c,** Image of the cells transfected with different plasmids as indicated. Arrows indicate pyroptotic cells. Scale bars, 20 μm. **d,** Comparison of LDH release-based cell death assay in the cells transfected with different plasmids as indicated. Mean ± s.d., two-tailed unpaired Student’s t-test. **e,** Image of the cells infected with different rAAVs as indicated. Arrows indicate pyroptotic cells. Scale bars, 20 μm. **f,** LDH release-based cell death assay of the cells infected with different rAAVs. Mean ± s.d., two-tailed unpaired Student’s t-test. All data are representative of three independent experiments. Source data are provided as a Source Data file.


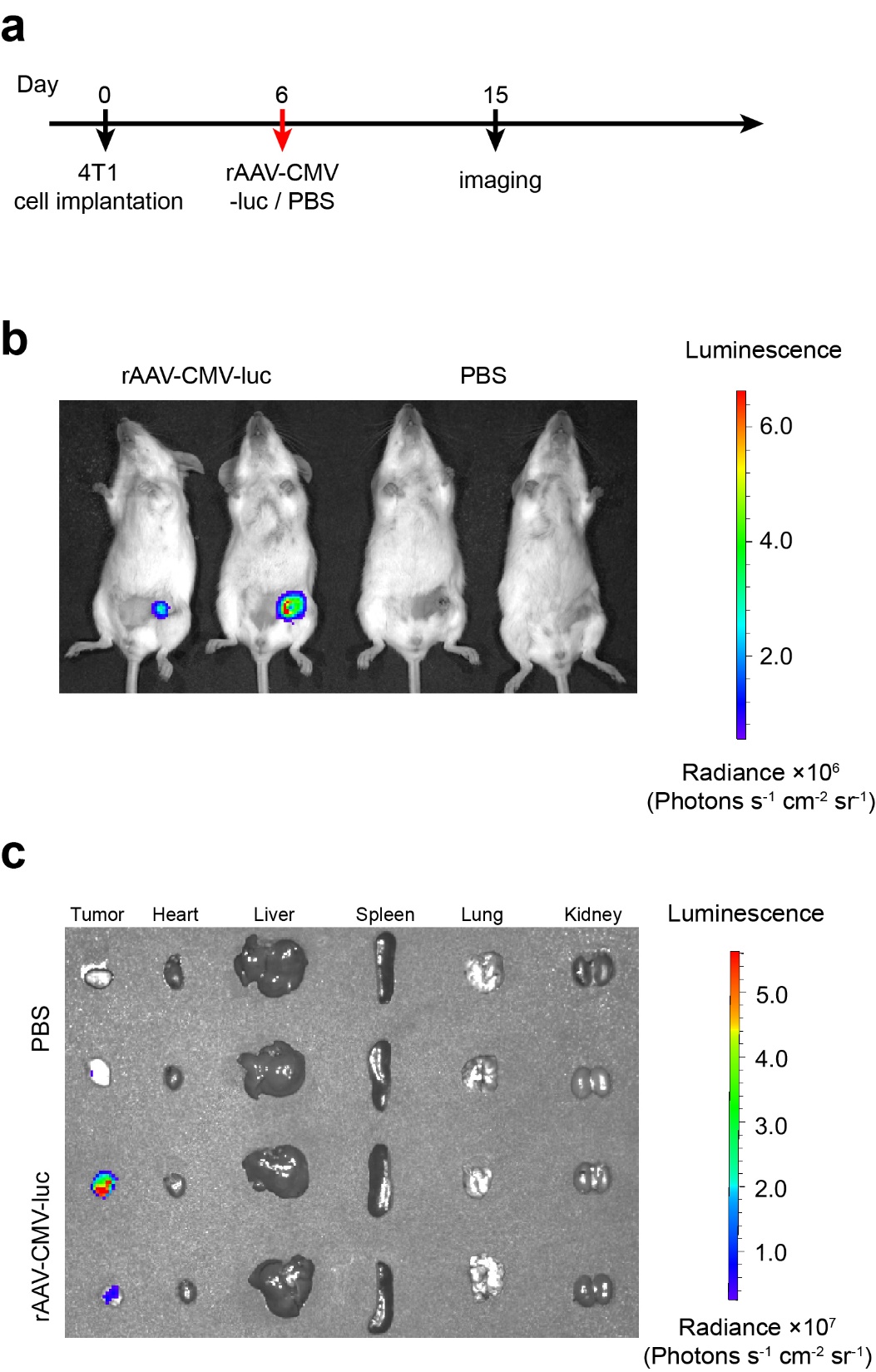


**Supplementary Figure 7. Evaluation of tumor targeting by rAAV intratumor injection.** **a,** Schematic of rAAV-CMV-luc treatment on TNBC mouse model. **b,** Luciferase imaging of mice in each group on day 9 post rAAV-CMV-luc or PBS intratumor injection, n = 2 mice for each group. **c,** Luciferase imaging of tumors and main organs on day 9 post rAAV-CMV-luc or PBS intratumor injection. All data are representative of two independent experiments.


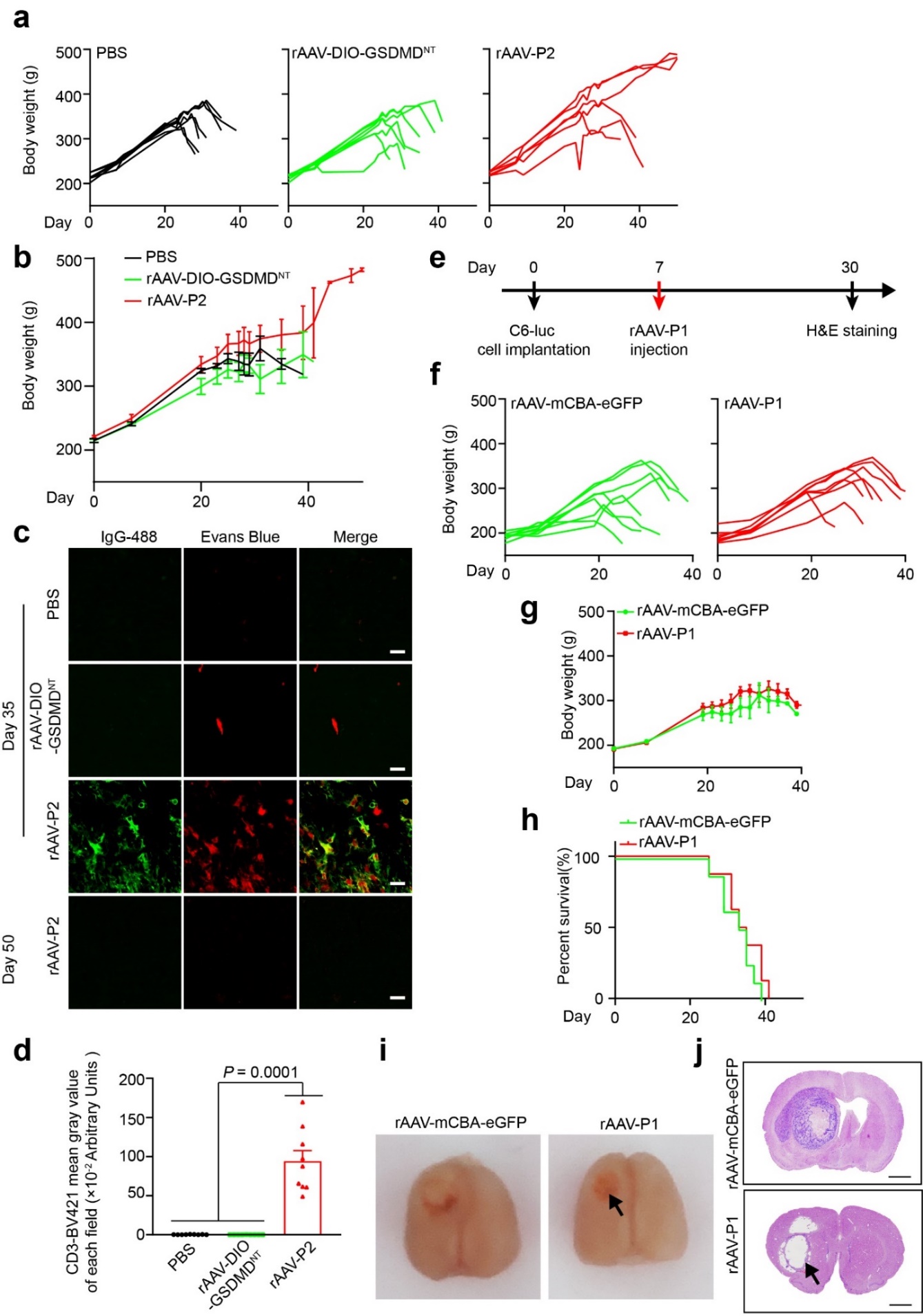


**Supplementary Figure 8. Effect of rAAV-P1 and rAAV-P2 treatments on Glioblastoma (GBM) rat model.** **a-d,** Effect of rAAV-P2 treatment on GBM rat model. **a, b,** Weight monitoring results of the rats as indicated, n = 7 rats for PBS and rAAV-mCBA-eGFP, n = 6 rats for rAAV-P2. **c,** Fluorescence images of IgG-488 and Evans Blue stained C6-luc tumors following treatment as indicated. Representative images of each group (n = 3 rats) are presented. Scale bar, 20 μm. **d,** Quantification of the tumor infiltrating CD3^+^ cells from GBM rat model by CD3-BV421 gray value measurement. Mean ± s.e.m., n=9 fields from n=3 individual rats., two-tailed unpaired Student’s t-test. **e-j,** rAAV-P1 treatment of GBM rat model. n = 8 rats for each group. **e,** Scheme of rAAV-P1 treatment of GBM rat model. **f, g,** Weight monitoring results of the rats as indicated. Data (**g**) were expressed as mean ± s.e.m. **h,** Survival of C6-luc tumor-bearing rat treated with rAAV-mCBA-eGFP or rAAV-P1 as indicated in **e**. **i,** Photographs of representative brains from GBM model on day 30 (Black arrow shows the striking cavitation). **j,** H&E-stained coronal sections of C6-luc tumor-bearing rat brains 30 d post tumor cell implantation. Representative images of each group (n = 3 rats) are presented. Scale bar, 2,000 μm. Date shown are representative of three (**a-d**) or two (**e-j**) independent experiments. Source data are provided as a Source Data file.


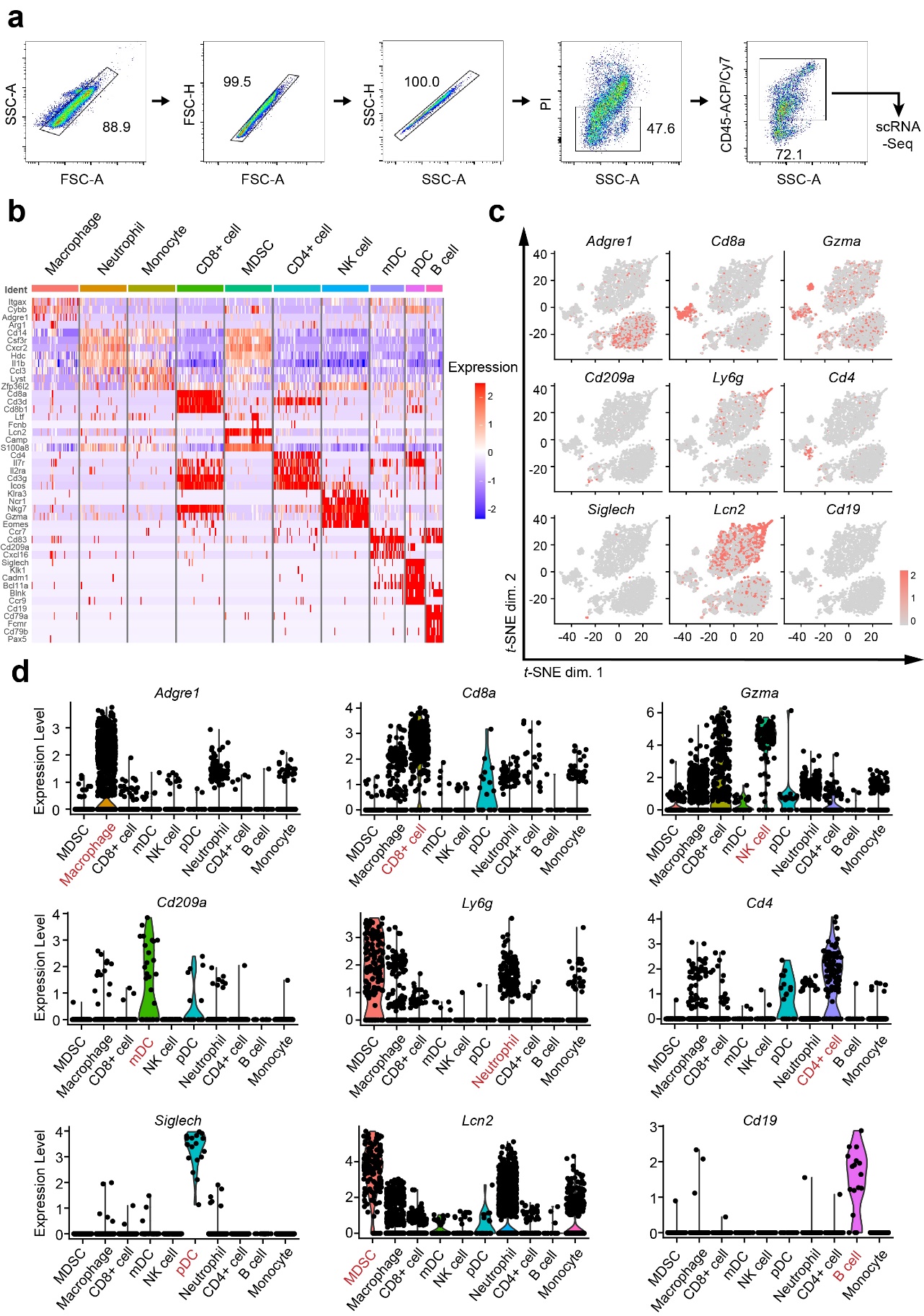


**Supplementary** **Figure 9. The single-cell transcriptome analysis of tumor infiltrating lymphocytes of TNBC mouse model treated with rAAV-P2.** **a,** Gating strategy and representative flowcytometry plots for the enrichment of 4T1 tumor-infiltrating single CD45^+^ immune cells**.** **b,** Heatmap of ten immune-cell clusters with unique signature genes. Colors on top of the map indicate the immune-cell clusters. The four or five marker genes used for each cluster are listed alongside the cluster. **c,** Signature gene-expression patterns for the corresponding cell clusters on the t-SNE plot (n = 4 mice, 5,159 cells). **d,** Violin plots show the expression of gene markers from the corresponding cell clusters. The cell cluster corresponding to each marker gene is marked in red.


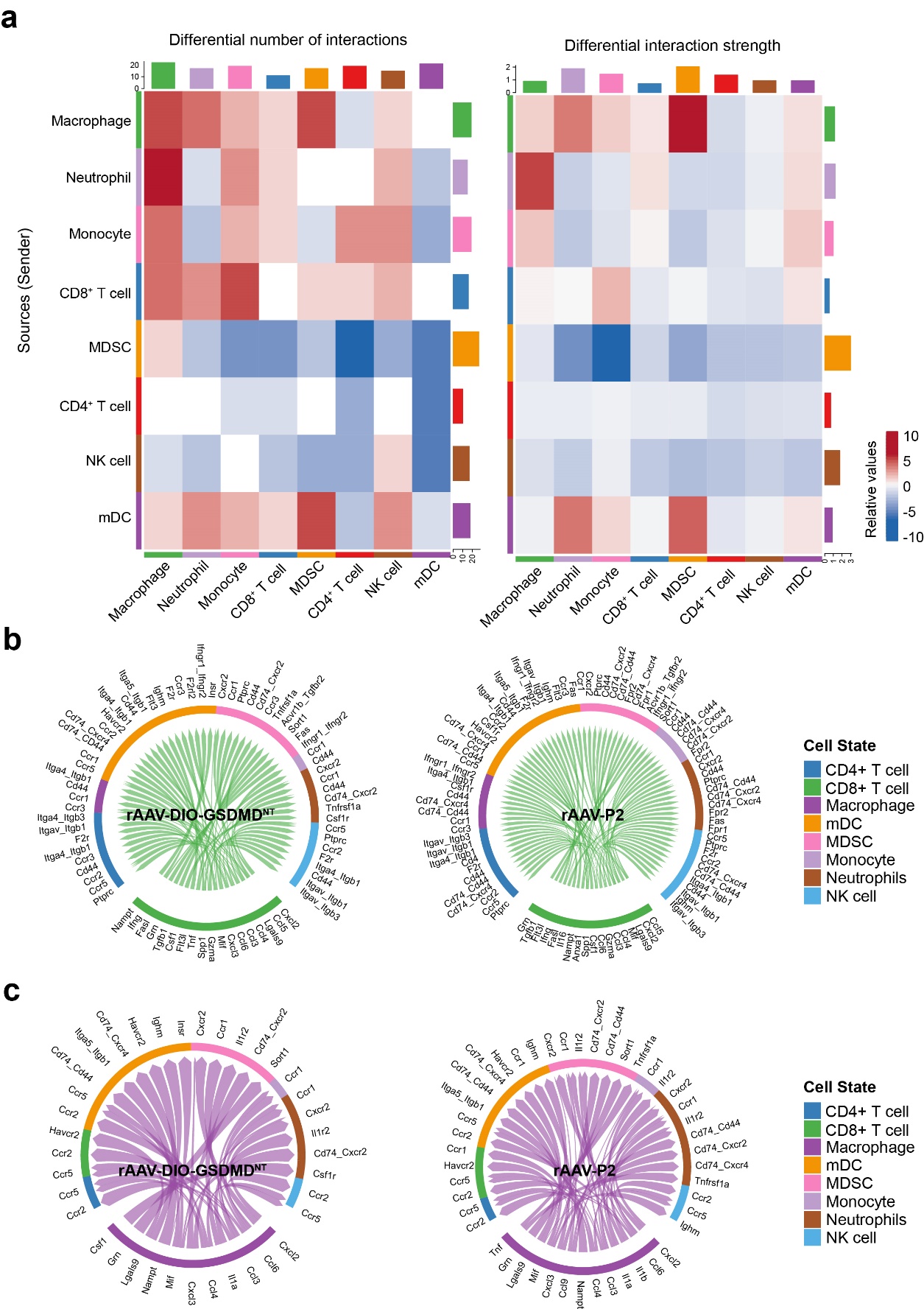


**Supplementary Figure 10. Illustrations of the integrated analysis of inter‐ and intracellular signaling.** **a,** The heatmap of differential number of interactions and interaction strength among different cell populations. The top-colored bar plot represents the sum of column of values displayed in the heatmap (incoming signaling). The right-colored bar plot represents the sum of row of values (outgoing signaling). **b,** Condition-specific linkages between CD8^+^ T cell ligands and other lymphocyte cluster receptors in rAAV-DIO-GSDMD^NT^ treatment group and rAAV-P2 treatment group. **c,** Condition-specific linkages between Macrophage ligands and other lymphocyte cluster receptors in rAAV-DIO-GSDMD^NT^ treatment group and rAAV-P2 treatment group.


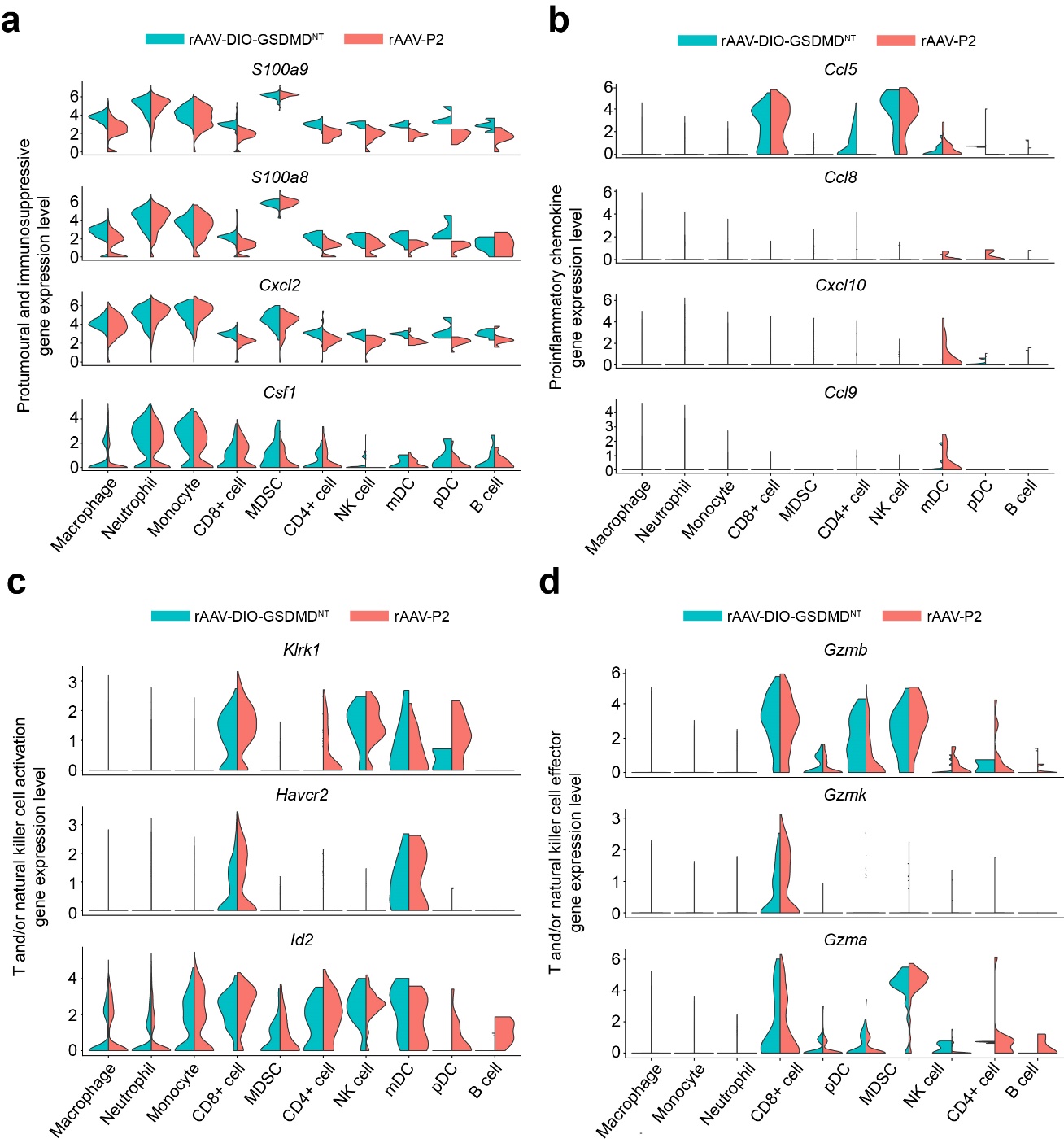


**Supplementary Figure 11. The single-cell transcriptome differential genes analysis of tumor infiltrating lymphocytes of TNBC mouse model treated with rAAV-P2.** **a-d,** The expression levels of protumoural and immunosuppressive genes (**a**), proinflammatory chemokines (**b**) and T and/or natural killer cell activation (**c**) or effector (**d**) genes in each immune cell cluster.


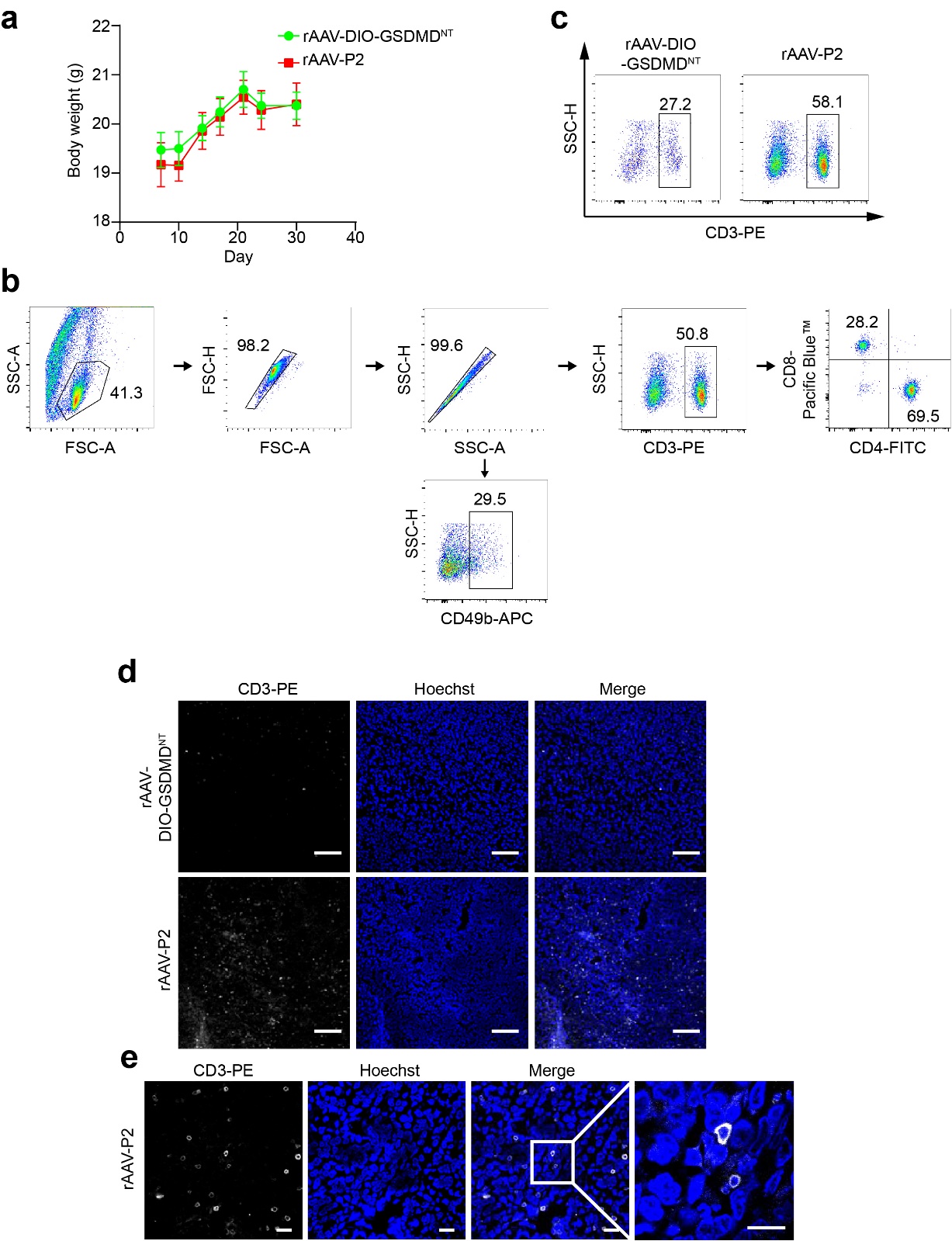


**Supplementary Figure 12. rAAV-P2 treatment activates anti-tumor immunity in TNBC mouse model.** **a,** Weight monitoring results of mice with different treatments as in Fig.4a. Data were expressed as mean ± s.e.m. **b, c,** Gating strategy (**b**) and representative flow cytometry plots for 4T1-luc tumor infiltrating CD3^+^ cells analysis (**c**). **d, e,** Fluorescence images of CD3-PE-stained 4T1-luc tumor cells treated with rAAV-DIO-GSDMD^NT^ (rAAV-ef1α-DIO-GSDMD^NT^) and rAAV-P2, respectively. Representative images of each group (n = 3 mice) are presented. Scale bar, 20 μm. Date shown are representative of three (**a, d, e**) or two (**c**) independent experiments. Source data are provided as a Source Data file.


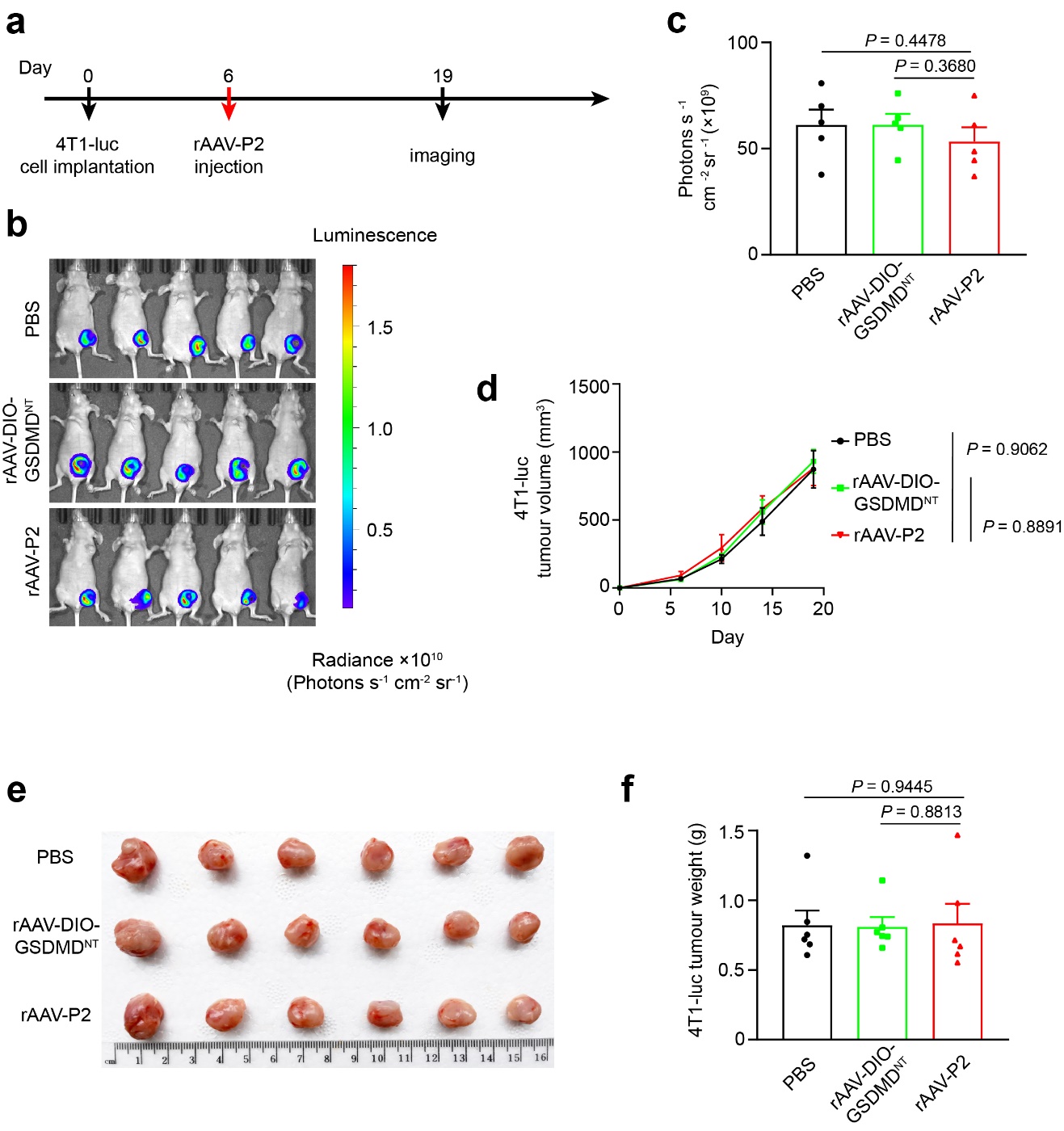


**Supplementary Figure 13.** **The rAAV-P2 treatment in TNBC Nude mouse model. a,** Schematic of rAAV-P2 treatment in TNBC nude mouse model. **b,** Luciferase imaging of 4T1-luc breast tumors 19 d post tumor implantation. **c,** Corresponding quantification of luciferase expression in **b**. Mean ± s.e.m., n = 5 mice for each group, two-tailed unpaired Student’s t-test. **d,** Average tumor volume of mice as indicated. n = 6 mice for PBS, AAV-DIO-GSDMD^NT^ (rAAV-ef1α-DIO-GSDMD^NT^) and AAV-P2. Data were expressed as mean ± s.e.m. Two-way ANOVA with the Geisser-Greenhouse correction was used for comparing different two groups. **e**, Photographs of representative tumors 19 d post treatment. **f,** Average tumor weight of mice as indicated. n = 6 mice per group. Mean ± s.e.m., two-tailed unpaired Student’s t-test. All date are representative of two independent experiments. Source data are provided as a Source Data file.


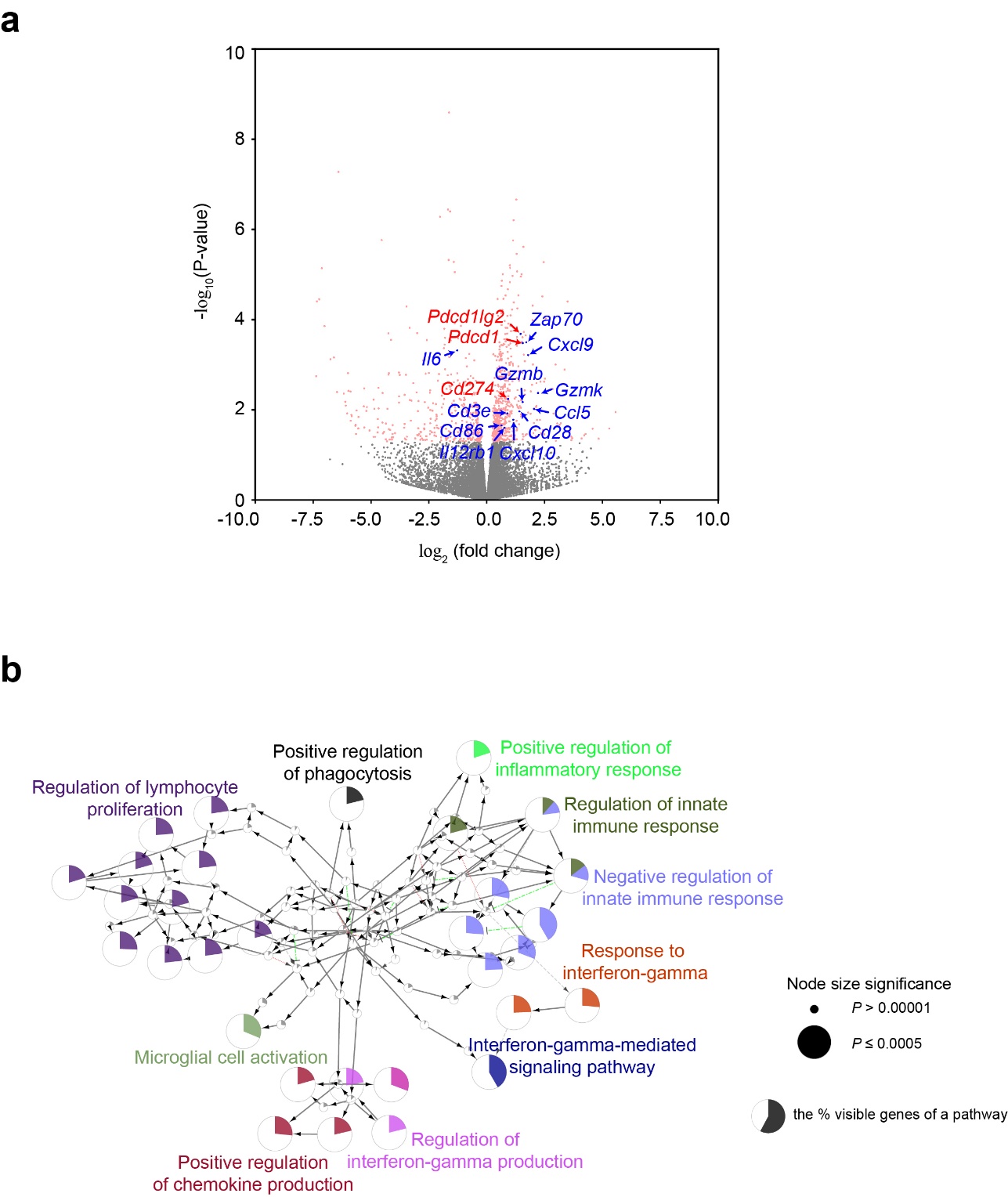


**Supplementary Figure 14. The transcriptome analysis of TNBC tumor tissue in mouse model treated with rAAV-P2. a,** Volcano plot of the differentially expressed genes. **b,** The biological process signal pathways network of GO enriched DEGs from Bulk RNA-seq of the tumors tissues in the rAAV-P2 group and rAAV-DIO-GSDMD^NT^ (rAAV-ef1α-DIO-GSDMD^NT^) group. *p* < 0.00001 (Two-sided hypergeometric test), Kappa Score = 0.5.


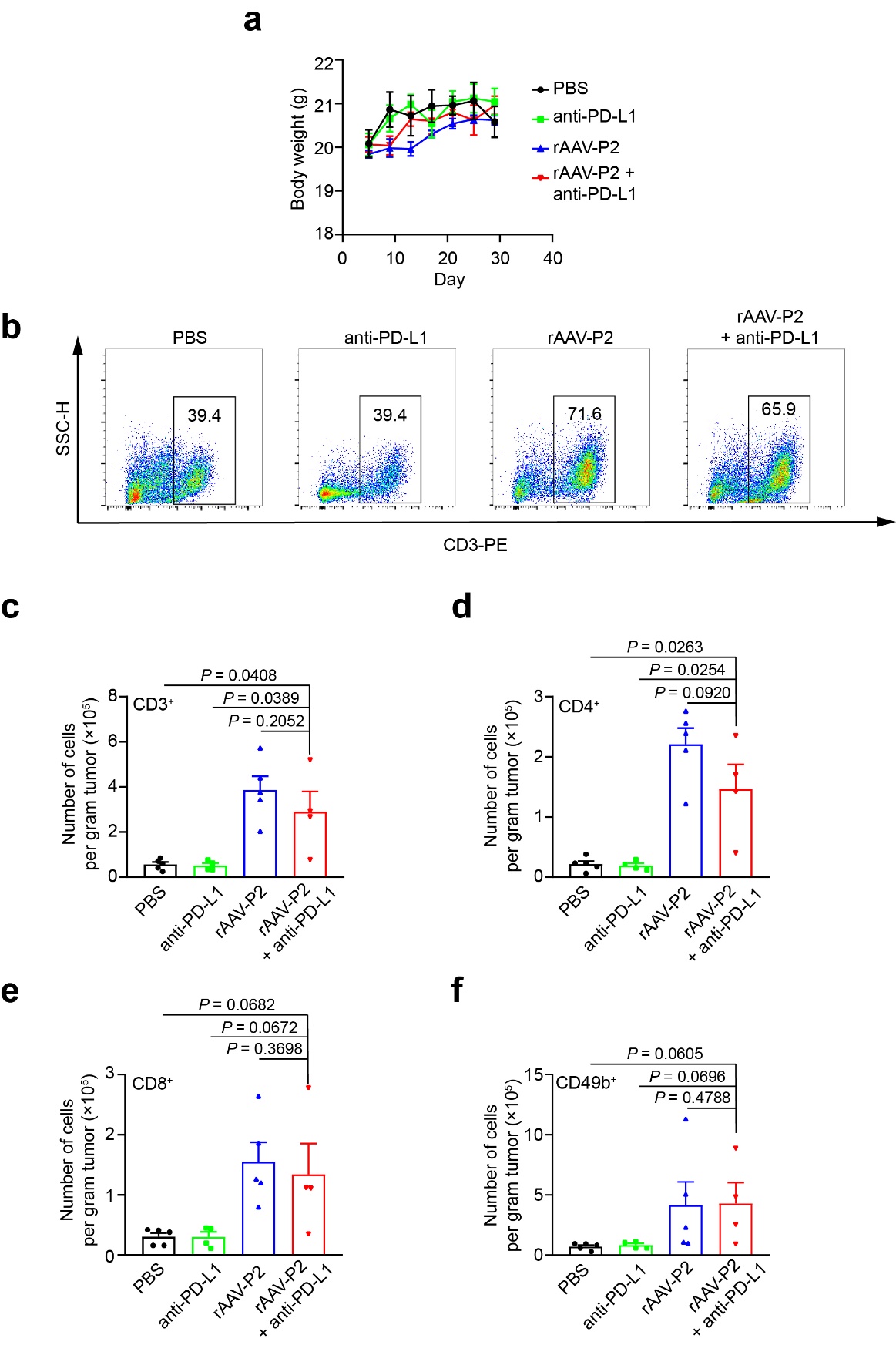


**Supplementary Figure 15. Anti-PD-L1 therapy boosts the effect of rAAV-P2 treatment on TNBC in mouse model. a,** Weight monitoring results of mice with different treatments as in Fig.5a. n = 5 mice per group. **b,** Representative flow cytometry plots for 4T1-luc tumor infiltrating CD3^+^ cells analysis. **c-f,** Quantification of the tumor infiltrating lymphocytes from TNBC mouse model with different treatments as in Fig.5a. n = 5 mice for PBS and rAAV-P2, 4 mice for anti-PD-L1 and rAAV-P2+anti-PD-L1. Mean ± s.e.m., one-tailed unpaired Welch’s t-test. Source data are provided as a Source Data file.


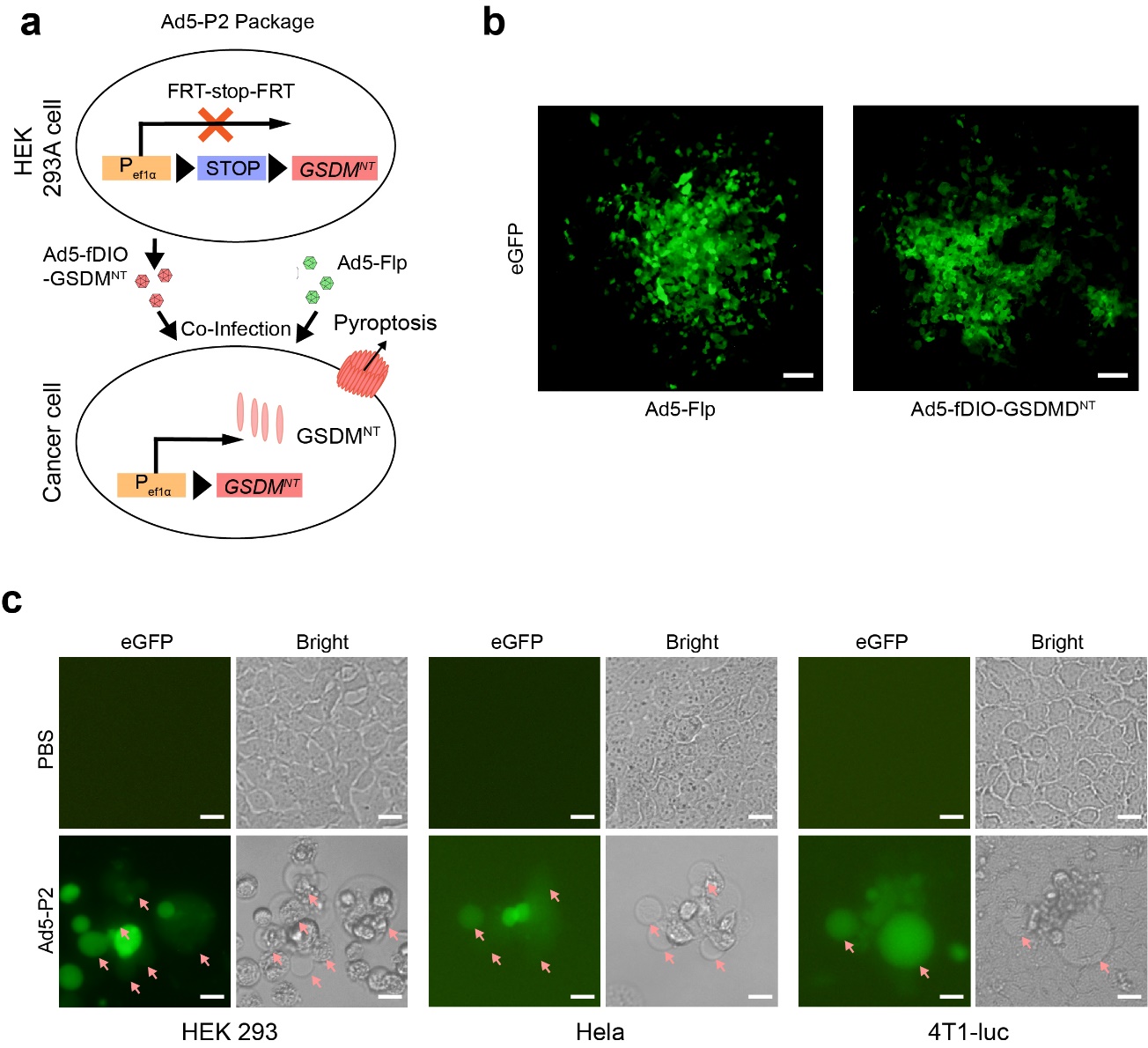


**Supplementary Figure 16. The Strategies for packaging Type 5 adenovirus vector (Ad5) expressing GSDMD^NT^ and the oncolytic effect analysis.** **a,** Schematic of the strategy using Flp/FRT system to package Ad5-fDIO-GSDMD^NT^. Co-infection of AAV-Cre can delete STOP (Ad5-P2) to induce pyroptosis. **b,** Representative images of fluorescent plaques formed by Ad5-Flp or Ad5-fDIO-GSDMD^NT^ infected HEK 293 cells. Scale bars, 20 μm. **c,** Images of HEK 293, Hela and 4T1-luc cells infected with Ad5-P2 (arrows indicate pyroptotic cells). Scale bars, 20 μm. Date shown (**b, c**) are representative of two independent experiments.


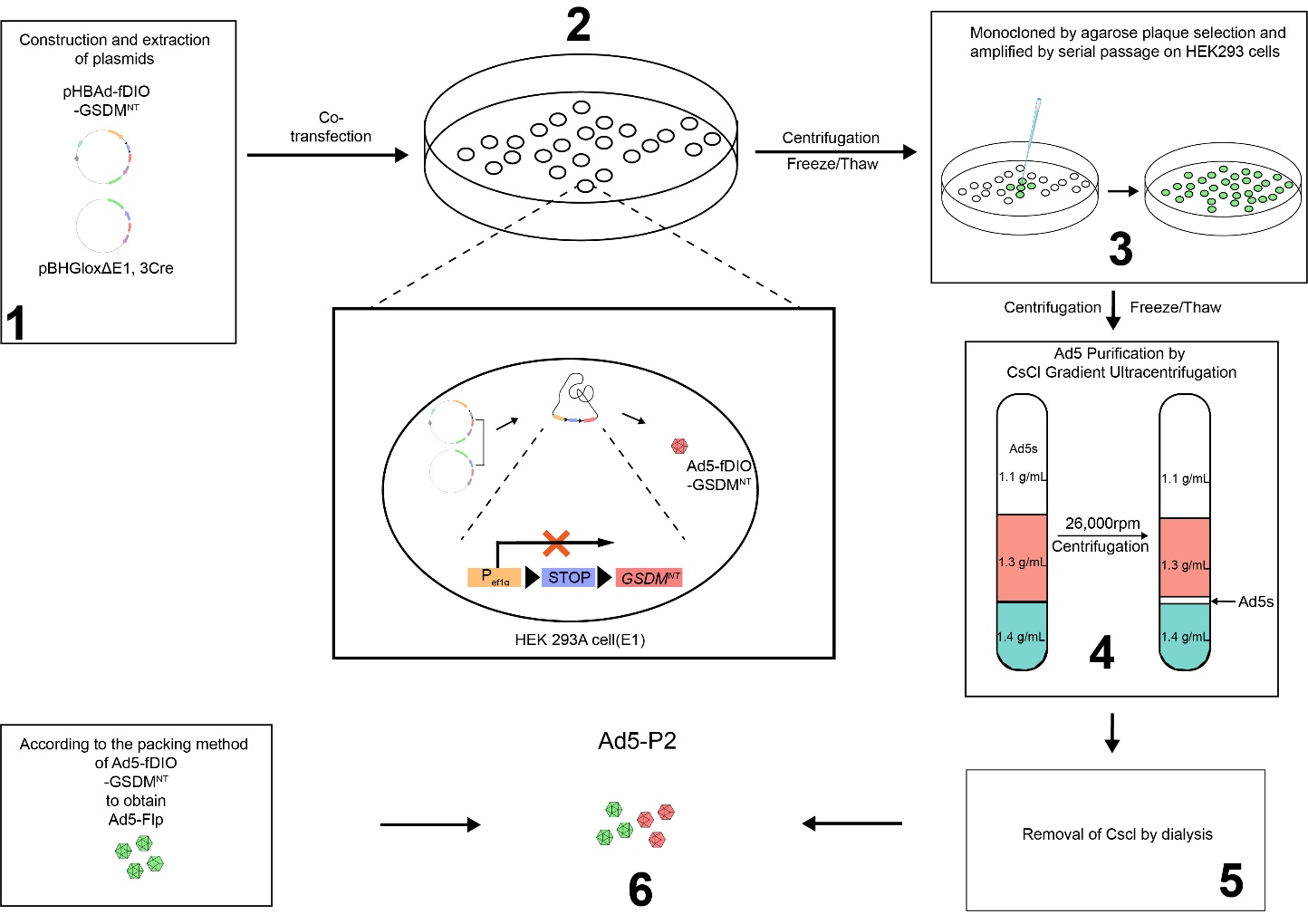


**Supplementary Figure 17. The flow diagram of Ad5-P2 packaging.** 1) Construction of plasmid pHBAd-fDIO-GSDM^NT^. 2) Co-transfect with “pBHGloxΔE1/3 Cre” into HEK 293 cells to package Ad5-fDIO-GSDM^NT^. FRT-STOP-FRT can avoid the expression of GSDM^NT^. 3) Monocloned by agarose plaque selection, amplified by serial passage on HEK 293 cells. 4) After CsCl density gradient centrifugation and 5) Concentration, Ad5-fDIO-GSDM^NT^ was obtained. 6) Ad5-Flp is also packaged to delete STOP for GSDM^NT^ expression. In this way, it can avoid the expression of GSDM^NT^ during virus packaging.


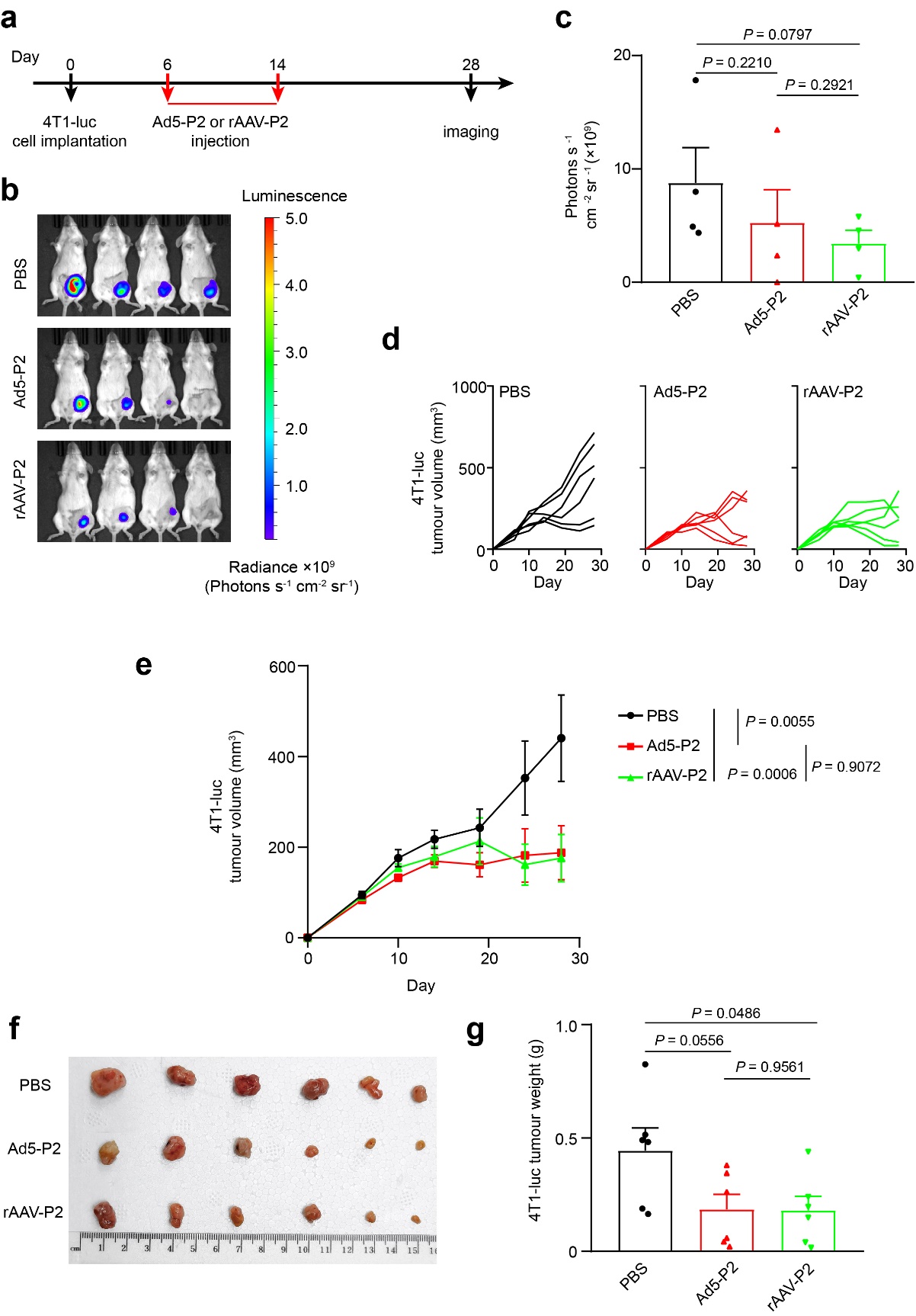


**Supplementary Figure 18. Ad5-P2 treatment on TNBC in mouse model. a,** Schematic of Ad5-P2 or rAAV-P2 treatment on TNBC mouse model. **b,** Luciferase imaging of 4T1-luc breast tumors 28 days post tumor implantation. **c,** Corresponding quantification of luciferase expression in **b**. Mean ± s.e.m., n = 4 mice for each group, one-tailed unpaired Student’s t-test. **d,** Tumor volume of an individual mouse. n = 6 mice per group. **e, g,** Average tumor volume (**e**) and weight (**g**) of mice as indicated. n = 6 mice per group. Data were expressed as mean ± s.e.m. Two-way ANOVA with the Geisser-Greenhouse correction was used for comparing different two groups (**e**). Two-tailed unpaired Welch’s t-test was used for comparing different two groups (**g**). **f,** Photographs of representative tumors 28 d post tumor implantation. All date are representative of two independent experiments. Source data are provided as a Source Data file.
