# Supplementary material for: Strategies to package recombinant Adeno-Associated Virus expressing the N-terminal gasdermin domain for tumor treatment
Source: Nat Commun. 2021 Dec 9;12:7155. doi: 10.1038/s41467-021-27407-0 (PMC8660823; doi:10.1038/s41467-021-27407-0)
Supplement: Supplementary file 2 — Reporting Summary [file 41467_2021_27407_MOESM2_ESM.pdf]

nature portfolio

Corresponding author(s): Gang Cao

Last updated by author(s): Nov 3, 2021

Reporting Summary

Nature Portfolio wishes to improve the reproducibility of the work that we publish. This form provides structure for consistency and transparency in reporting. For further information on Nature Portfolio policies, see our Editorial Policies and the Editorial Policy Checklist.

Statistics

For all statistical analyses, confirm that the following items are present in the figure legend, table legend, main text, or Methods section.

n/a	Confirmed	
☐	☒	The exact sample size (n) for each experimental group/condition, given as a discrete number and unit of measurement
☐	☒	A statement on whether measurements were taken from distinct samples or whether the same sample was measured repeatedly
☐	☒	The statistical test(s) used AND whether they are one- or two-sided<br>*Only common tests should be described solely by name; describe more complex techniques in the Methods section.*
☒	☐	A description of all covariates tested
☐	☒	A description of any assumptions or corrections, such as tests of normality and adjustment for multiple comparisons
☐	☒	A full description of the statistical parameters including central tendency (e.g. means) or other basic estimates (e.g. regression coefficient) AND variation (e.g. standard deviation) or associated estimates of uncertainty (e.g. confidence intervals)
☐	☒	For null hypothesis testing, the test statistic (e.g. F, t, r) with confidence intervals, effect sizes, degrees of freedom and P value noted<br>*Give P values as exact values whenever suitable.*
☒	☐	For Bayesian analysis, information on the choice of priors and Markov chain Monte Carlo settings
☒	☐	For hierarchical and complex designs, identification of the appropriate level for tests and full reporting of outcomes
☒	☐	Estimates of effect sizes (e.g. Cohen's d, Pearson's r), indicating how they were calculated

Our web collection on statistics for biologists contains articles on many of the points above.

Software and code

Policy information about availability of computer code

Data collection	PerkinElmer Living Image® (IVIS Spectrum), PerkinElmer Harmony 4.9 (Opera Phenix™ High Content Screening System), Olympus FV10-ASW 3.1 (FV1000), Beckman Coulter CytExpert (Cytoflex LX)
Data analysis	PerkinElmer Living Image® (IVIS Spectrum), Olympus FV10-ASW 3.1 (FV1000), nf-core rnaseq(1.4.2), DEseq2(1.16.1), GraphPad Prism version 8, Seurat (v.3.2.3), CellChat (1.1.0)

For manuscripts utilizing custom algorithms or software that are central to the research but not yet described in published literature, software must be made available to editors and reviewers. We strongly encourage code deposition in a community repository (e.g. GitHub). See the Nature Portfolio guidelines for submitting code & software for further information.

Data

Policy information about availability of data

All manuscripts must include a data availability statement. This statement should provide the following information, where applicable:

- Accession codes, unique identifiers, or web links for publicly available datasets
- A description of any restrictions on data availability
- For clinical datasets or third party data, please ensure that the statement adheres to our policy

scRNA-Seq and bulk RNA-Seq data that support the findings of this study have been deposited at the National Genomics Data Center (NGDC) under accession number PRJCA005365 (https://bigd.big.ac.cn/bioproject/browse/PRJCA005365) and PRJCA003564 (https://bigd.big.ac.cn/bioproject/browse/PRJCA003564). Source Data for Figs. 1–5, Supplementary Figs. 2, 5, 6, 8, 12, 13, 15, 18 are available with the paper. All other data supporting the findings of this study are available from the corresponding author upon reasonable request.

Field-specific reporting

Please select the one below that is the best fit for your research. If you are not sure, read the appropriate sections before making your selection.

- [x] Life sciences
- [ ] Behavioural & social sciences
- [ ] Ecological, evolutionary & environmental sciences

For a reference copy of the document with all sections, see nature.com/documents/nr-reporting-summary-flat.pdf

Life sciences study design

All studies must disclose on these points even when the disclosure is negative.

Sample size	No statistical methods were used to predetermine sample size. On the basis of achieving statistical significance ($p < 0.05$), we selected the minimum number of animals (at least three animals per treatment group) for the group sizes.
Data exclusions	No exclusion of data was made.
Replication	All experimental data was reliably reproduced in multiple independent experiments as indicated in the figure legends.
Randomization	For all experiments, animals or cells were randomly divided into treatment groups after tumor inoculation. Before treatment, the initial tumor burden of the treatment group and the control group were similar.
Blinding	Measurement of the tumor sizes and animal weights were performed by a person blinded to the treatment groups. Other experiments cannot be blinded due to objective factors, but this does not affect the conclusion of the article.

Reporting for specific materials, systems and methods

We require information from authors about some types of materials, experimental systems and methods used in many studies. Here, indicate whether each material, system or method listed is relevant to your study. If you are not sure if a list item applies to your research, read the appropriate section before selecting a response.

Materials & experimental systems

n/a	Involved in the study	
☐	☒	Antibodies
☐	☒	Eukaryotic cell lines
☒	☐	Palaeontology and archaeology
☐	☒	Animals and other organisms
☒	☐	Human research participants
☒	☐	Clinical data
☒	☐	Dual use research of concern

Methods

n/a	Involved in the study	
☒	☐	ChIP-seq
☐	☒	Flow cytometry
☒	☐	MRI-based neuroimaging

Antibodies

Antibodies used	Anti-Rat antibody CD3-BV421 (clone 1F4) was purchased from BD Biosciences (United States of America). Anti-rat IgG-488 was purchased from Invitrogen (United States of America). Antibodies for flow cytometry analysis, CD3-PE (clone 17A2), CD4-FITC (clone RM4.5), CD8a-PacBlue (clone 53-6.7), CD49b-APC (clone DX5) and CD45-APC-Cy7 (clone 30-F11) were purchased from BioLegend (United States of America).
Validation	All antibodies were well-recognized clones in the field and validated by the manufacturers. These antibodies are further validated and routinely used in our lab.<br>Anti-Rat antibody CD3-BV421 (Cat # 563948, clone 1F4) https://www.bdbiosciences.com/cn/applications/research/t-cell-immunology/th-1-cells/surface-markers/rat/bv421-mouse-anti-rat-cd3-1f4/p/563948<br>Anti-rat IgG-488 (Cat # A48262) https://www.thermofisher.cn/cn/zh/antibody/product/Goat-anti-Rat-IgG-H-L-Highly-Cross-Adsorbed-Secondary-Antibody-Polyclonal/A48262.<br>CD3-PE (Cat # 100205, clone 17A2) https://www.biolegend.com/en-us/products/pe-anti-mouse-cd3-antibody-47<br>CD4-FITC (Cat # 100509, clone RM4.5) https://www.biolegend.com/en-us/products/fitc-anti-mouse-cd4-antibody-480<br>CD8a-PacBlue (Cat # 100728, clone 53-6.7) https://www.biolegend.com/en-us/products/pacific-blue-anti-mouse-cd8a-antibody-2856<br>CD49b-APC (Cat # 108909, clone DX5) https://www.biolegend.com/en-us/products/apc-anti-mouse-cd49b-pan-nk-cells-antibody-231<br>CD45-APC-Cy7 (Cat # 103115, clone 30-F11) https://www.biolegend.com/en-us/search-results/apc-cyanine7-anti-mouse-cd45-antibody-2530

Eukaryotic cell lines

Policy information about cell lines

Cell line source(s)	Hela and HEK 293T cells were obtained from the American Type Culture Collection (ATCC). Hep3B and sf9 cells were gifted by Professor Mingqian Feng (Huazhong Agricultural University) and Professor Guiqing Peng respectively and both cells were purchased from ATCC. 4T1 and C6 cells were purchased from the Cell Resource Center of Shanghai Institutes for Biological Sciences, Chinese Academy of Sciences. The 4T1-luc and C6-luc cell lines expressing luciferase were selected from 4T1 and C6 cells after infection with lentivirus-luc.
Authentication	The cell lines were routinely authenticated by growth curve analysis and morphology check using microscope.
Mycoplasma contamination	All cell lines were assessed regularly to ensure they were free of mycoplasma contamination.
Commonly misidentified lines (See ICLAC register)	No commonly misidentified cell lines were used.

Animals and other organisms

Policy information about studies involving animals; ARRIVE guidelines recommended for reporting animal research

Laboratory animals	Balb/c female mice, 6-8 week-old, 20±2g; Wistar male rats, 7 week-old, 200±2g; Nu/Nu nude female mice, 6-8 week-old, 20±2g
Wild animals	The study did not involve wild animals.
Field-collected samples	The study did not samples collected from the filed.
Ethics oversight	All animal studies were performed in accordance with the Guide for the Care and Use of Laboratory Animals of the Research Ethics Committee of Huazhong Agricultural University. The use of mice and rats has been approved by the Research Ethics Committee of Huazhong Agricultural University, Hubei, China (HZAUMO-2019-041).

Note that full information on the approval of the study protocol must also be provided in the manuscript.

Flow Cytometry

Plots

Confirm that:

- [x] The axis labels state the marker and fluorochrome used (e.g. CD4-FITC).
- [x] The axis scales are clearly visible. Include numbers along axes only for bottom left plot of group (a 'group' is an analysis of identical markers).
- [x] All plots are contour plots with outliers or pseudocolor plots.
- [x] A numerical value for number of cells or percentage (with statistics) is provided.

Methodology

Sample preparation	The mice were anesthetized and sacrificed to separate tumor masses. The tumors were cut into pieces and ground, then filtered through a 70-μm cell sieve to obtain a single cell suspension. Lymphocytes were separated with The Mouse Tumor Infiltrating Tissue Lymphocyte Separation Kit (Solarbio).
Instrument	Cytoflex LX (Beckman Coulter)
Software	Beckman Coulter CytExpert(Cytoflex LX)
Cell population abundance	Lymphocytes were separated with The Mouse Tumor Infiltrating Tissue Lymphocyte Separation Kit (Solarbio).
Gating strategy	Cell were first gated in intact cells using FSC/SCC, doublets were excluded using FSC-H vs FSC-A and SSC-H vs SSC-A, cells were followed by cell type specific gating using fluorescently labeled antibodies.

- [x] Tick this box to confirm that a figure exemplifying the gating strategy is provided in the Supplementary Information.
